# Properties and Stability of Perilla Seed Protein-Stabilized Oil-in-Water Emulsions: Influence of Protein Concentration, pH, NaCl Concentration and Thermal Treatment

**DOI:** 10.3390/molecules23071533

**Published:** 2018-06-26

**Authors:** Ning Liu, Qiannan Chen, Guanghui Li, Zhenbao Zhu, Jianhua Yi, Cheng Li, Xuefeng Chen, Yong Wang

**Affiliations:** 1 School of Food and Biological Engineering, Shaanxi University of Science and Technology, Xi’an 710021, China; 13703869157@163.com (Q.C.); zhuzhenbao@sust.edu.cn (Z.Z.); yijianhua@sust.edu.cn (J.Y.); dthaowen@163.com (C.L.); chenxf@sust.edu.cn (X.C.); 2 Department of Food Science and Engineering, College of Science and Engineering, Jinan University, Guangzhou 510632, China; bruce@2015stu.jnu.edu.cn

**Keywords:** perilla seed protein, O/W emulsion, pH, NaCl concentration, thermal treatment

## Abstract

Perilla seed protein (PSP) was extracted from defatted perilla seed meal and applied in oil-in-water (O/W) emulsions as an emulsifier. We investigated the influences of protein concentration (0.25–1.5 wt %), pH (3.0–9.0), NaCl concentration (0–350 mmol/L) and thermal treatment (70–90 °C, 30 min) on the physical characteristics of O/W emulsions, including volume-average diameter, ζ-potential, interfacial protein concentration, microstructure and so on. Results showed that increasing PSP concentration would decrease the *d*_4,3_ and a 1.0 wt % PSP concentration was sufficient to ensure the stability of emulsion. Under pH 3.0–9.0, emulsions were stable except at pH 3.0–5.0 which was proximal to the isoelectric point (pH 4.5) of PSP. At high NaCl concentrations (250–350 mmol/L), the emulsions exhibited relatively lower absolute ζ-potential values and a large number of aggregated droplets. A moderate thermal treatment temperature (e.g., 70 °C) was favorable for the emulsion against aggregation and creaming. However, when 90 °C thermal treatment was performed, a clear layer separation was observed after 2 weeks storage and the emulsion showed a poor stability. The findings of this work are of great importance for the utilization and development of PSP as an emulsifier for food emulsions.

## 1. Introduction

Perilla (*Perilla frutescens*) belongs to the family *Lamiaceae*, which is an edible plant and often used as garnishes and food pigments in Japan and China [1]. It is also brought into Europe, Russia and USA as an oil seed crop [2]. Perilla seed meal, originated from the extraction of perilla seed oil, is a vital agricultural outgrowth, because of its enriched content of proteins, phenolic compounds, phytic acid and polysaccharides. The utilization and development of these compounds have received major attention recently to increase the rentability of perilla as an agricultural goods [3,4]. Takenaka et al. [5] found the defatted perilla seed meal contained approximately 40% protein, thereunto the globulin was divided into three intermediary subunits (54, 57 and 59 kDa) by SDS-PAGE.

Nowadays, consumers are pursuing food products with natural additives and this demand highlights the importance of investigating the effectiveness of the natural emulsifiers in food emulsions [6,7]. Protein is a kind of emulsifier widely used in food emulsions attributing to their amphipathic property. They can stabilize emulsions by adsorbing at the interface of the droplets and then reducing the interfacial tension. Moreover, films formed by proteins at the interface can provide electrostatic repulsion and steric hindrance between droplets [8]. Owing to the prominent surface-active and emulsion stabilizing properties that dairy proteins shows, they are usually used as emulsifiers in food industry [9]. However, due to the high cost of dairy ingredients, there is an increasing interest in utilizing plant proteins as an alternative for dairy proteins. Emulsion-based products often experience multifarious environments during processing such as presence of salts and acids [10]. Furthermore, food commodity containing fat droplets may be subjected to all sorts of thermal treatments during their manufacture, storage and utilization, for example, pasteurization, temperature changes and cooking [11].

Many researchers have examined the characteristics of oil-in-water (O/W) emulsions stabilized by varieties of plant proteins. Guo and Mu [12] studied the effect of sweet potato protein concentration and oil volume fraction on the emulsifying properties. Their results suggested that droplet aggregation was observed at a lower protein concentration of less than 0.5% (*w*/*v*) because of low interfacial protein concentration. Moreover, at higher oil volume fractions of more than 25% (*v*/*v*) there was evident coalescence. Shao and Tang [13] investigated the properties of O/W emulsions stabilized by soy protein and they found that increasing protein concentration was beneficial to the emulsification efficiency but the flocculated condition of oil droplets or size of the flocs in the fresh emulsions was more influenced by the presence of NaCl (e.g., 300 mmol/L), and/or the thermal pretreatment (e.g., 95 °C, 15 min). Sarkar et al. [10] examined the stability of O/W emulsions stabilized by tomato seed protein isolate. They found that emulsions showed remarkable stability to high NaCl concentrations (250 mol/L) at pH 6.0–8.0 and droplet aggregation in emulsions occurred at above 80 °C. The influence of NaCl on the oil/water interface and emulsifying characteristics of walnut protein-xanthan gum in O/W emulsions were examined by Tan et al. [14]. Their results indicated that 150–500 mmol/L NaCl would decrease ζ-potential and aggravate flocculation, as large droplets were observed at 500 mmol/L NaCl.

Taking into account the previous works did not evaluate the application of PSP in O/W emulsions, in the present work, the influences of protein concentration, pH, salt concentration and thermal treatment on the physical properties of PSP stabilized-emulsions will be studied.

## 2. Results and Discussion

### 2.1. Effect of PSP Concentration on the Physical Properties of Emulsions

PSP dispersions were firstly prepared and a RVA-TecMaste (Perten Instruments, Stockholm, Sweden) was used to determine the viscosity. As to the PSP dispersions of 0.25–1.5 wt % concentration, the viscosity values were in the range of 6.05–8.15 Pa·s. The ζ-potential of PSP in 1.0 wt % dispersion was measured as −32.36 ± 0.07 mV.

The O/W emulsions based on PSP were then prepared. The effect of PSP concentration (0.25–1.5 wt %) on the physical properties (*d*_4,3_, ζ-potential, interfacial protein concentration) of emulsions were determined and the results are shown in Table 1. When 0.25 wt % protein concentration used, the *d*_4,3_ was the largest (5.54 μm). Increasing the protein concentration (up to 1.5 wt %) would decrease the *d*_4,3_. There was no significant difference (*p* > 0.05) between samples of 1.0 wt % and 1.5 wt % PSP concentration. Moreover, increasing PSP concentration led to corresponding increase in absolute ζ-potential value. When protein concentration increased from 0.25 wt % to 1.5 wt %, it reached −49.34 mV. It could be also found that there was no significant difference (*p* > 0.05) between emulsions with 1.0 wt % and 1.5 wt % protein concentrations. The ζ-potential is a physical index measuring the intensity of electrostatic repulsion between particles in suspension system [15]. Usually, the higher the absolute ζ-potential value, the more stable the system is [13].

The main reason for the reduce in *d*_4,3_ with increased protein concentration is protein surface-coverage, which can be described as interfacial protein concentration. The *d*_4,3_ of droplets of the dispersed oil phase is generally determined by the balance of droplet disruption and coalescence during emulsification. The high *Γ* promotes droplet disruption and prevents droplet coalescence within the homogenizer, which leads to smaller *d*_4,3_ [12]. While PSP concentration increased from 0.25 wt % to 1.0 wt %, the *Γ* increased from 0.65 mg/m^2^ to 1.55 mg/m^2^ significantly (*p* < 0.05). There was no significant difference (*p* > 0.05) between emulsions with 1.0 wt % and 1.5 wt % protein concentrations. The *Γ* is a crucial parameter affecting the ability of oil droplets against coalescence. In usual, the higher the *Γ* is, the greater the emulsion ability against coalescence [13]. Therefore, in this work, we inferred that at lower PSP concentrations, there were not enough proteins adsorbed at the interface. Overall, a 1.0 wt % protein concentration was enough to cover the surface of oil droplets.

### 2.2. Effect of pH on the Physical Properties of PSP-Stabilized Emulsions

**Figure 1:**
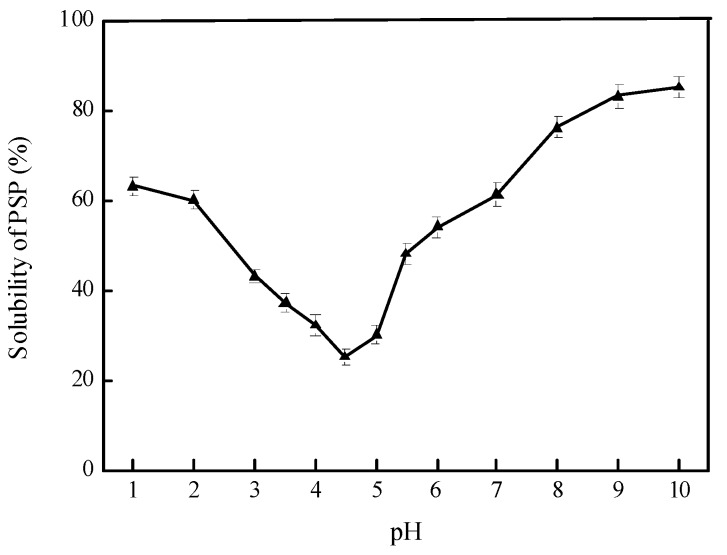
pH-solubility curve of Perilla seed protein (PSP). Values are means ± SDs (n = 3).

Figure 1 shows the pH-solubility curve of PSP. The results illustrated that PSP had the lowest solubility at pH 4.5 and the highest solubility at pH 10.0. Generally, the solubility of proteins decreases at pH around its isoelectric point. At this point, the net charge of proteins is near zero and the proteins is prone to aggregate due to the electrostatic interactions caused by the charge asymmetry of the proteins [16,17].

**Figure 2:**
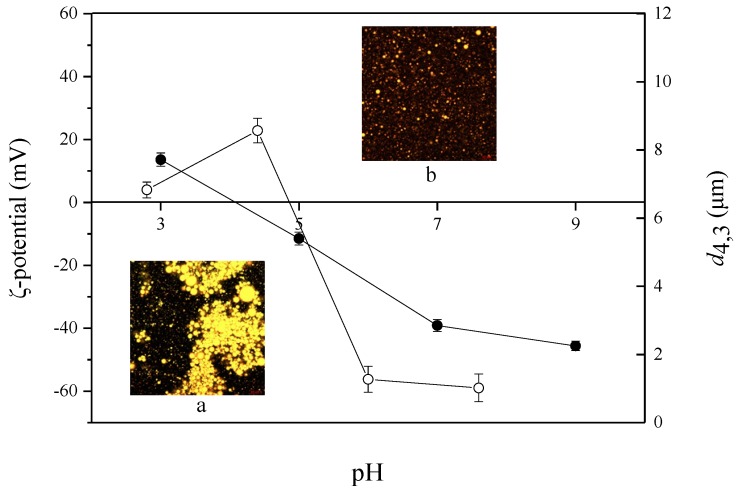
Influence of pH on ζ-potential (●) and *d*_4,3_ (○) of PSP-stabilized emulsions. Insets show the confocal laser scanning microscopy (CLSM) microstructures at (**a**) pH 5.0, (**b**) pH 7.0. Scale bar in the micrographs corresponds to 10 μm. Values are means ± SDs (n = 3).

The stabilization of emulsions against aggregation and flocculation is mainly relied on the repulsive forces between droplets, which might shift based on pH change [18]. Emulsions with different pH (3.0–9.0) were prepared and their physical properties are shown in Figure 2. As pH increased from 3.0 to 5.0, the *d*_4,3_ increased and the ζ-potential values ranged between 13.63 mV and −11.52 mV. Such low extent of absolute ζ-potential values suggested that the emulsions being at or close to the isoelectric point (pH 4.5, Figure 1) of PSP. This may influence the conformation of molecular and therefore the functional properties of molecules, by shielding electrostatic repulsion, binding to negatively charged groups and thus decreasing the number of similarly charged groups [19]. Therefore, the electrostatic interactions of the PSP adsorption layers were unable to avoid the droplets aggregating together [8], which could be observed clearly in the confocal laser scanning microscopy (CLSM) graph (Figure 2a).

At pH above 5.0, the *d*_4,3_ decreased and the absolute ζ-potential values increased, indicating that the net negative charge increased to stabilize the droplets against aggregation. The CLSM graph of emulsion at pH 7.0 (Figure 2b) supported this. As expected, above the isoelectric point, the carboxyl groups would become negatively charged and the amino groups is neutral which in turn generate net negative charges among droplets. Therefore, in protein-stabilized emulsions, the most probable stabilization mechanism preventing droplet aggregation is electrostatic interactions [10]. These results indicated that PSP was potential to be applied in O/W emulsions in neutral or alkaline environment.

### 2.3. Effect of Salts on the Physical Properties of PSP-Stabilized Emulsions

**Figure 3:**
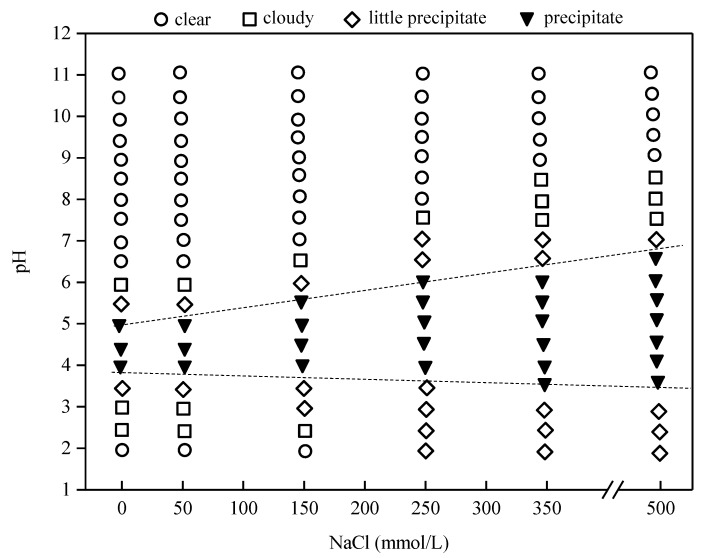
Phase diagram of PSP in aqueous solution.

Salt is a common ingredient in many food emulsions, such as milk, butter, cream, soups, margarine, sauces and mayonnaise [20]. Figure 3 is the effect of NaCl concentration and pH on the phase behavior of PSP dispersions. It can be observed that with the increase of NaCl concentration, phase separation (precipitate) occurred in these dispersions. Moreover, this phase separation region became wider and shifted towards high pH values and the stability of the system decreased. This was due to the screening effect of NaCl, as the electrostatic repulsion between proteins in the dispersions could be weakened and this effect would aggravate with the increase of NaCl concentration.

**Figure 4:**
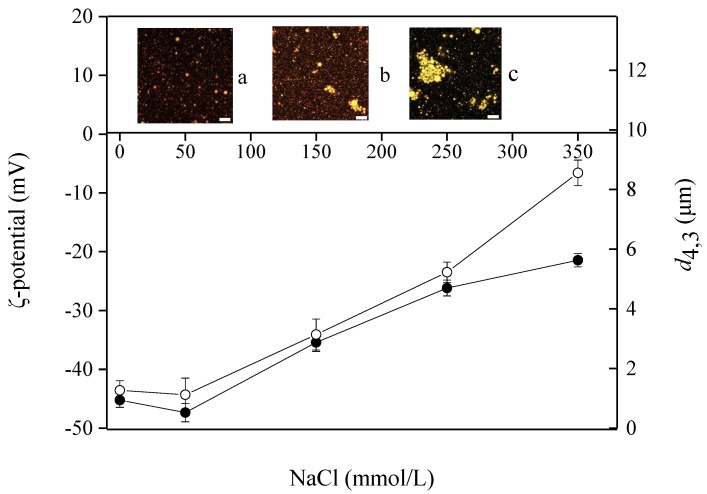
Influence of salt concentration on ζ-potential (●) and *d*_4,3_ (○) of PSP-stabilized emulsions. Insets show the CLSM microstructures at (**a**) 50 mmol/L NaCl, (**b**) 150 mmol/L NaCl and (**c**) 250 mmol/L NaCl. Scale bar in the micrographs corresponds to 10 μm. Values are means ± SDs (n = 3).

The effects of salt concentration on the physical characteristics of emulsions (pH 7.0) were examined and the results are shown in Figure 4. At 50 mmol/L NaCl concentration, the *d*_4,3_ was 1.12 μm, which did not show significant difference (*p* > 0.05) compared with the control sample. When 350 mmol/L NaCl concentration involved, the maximum *d*_4,3_ value was observed (8.57 μm). Meanwhile, this sample showed the lowest absolute ζ-potential value (23.35 mV). Figure 4a–c shows the CLSM graphs of PSP emulsions with 50, 150 and 250 mmol/L NaCl concentration, respectively. It could be clearly found that 250 mmol/L NaCl concentrations resulted in droplet flocculation to some extent and several clustered and irregular flocs were observed.

Emulsions are apt to flocculate when the salts concentration exceeds a particular level, because the repulsive forces between the droplets are relatively weak to overcome the attractive forces [10]. In the present work, this particular level should be between 150–250 mmol/L NaCl concentration. Previous studies on protein stabilized-O/W emulsions revealed that the main stabilizing force between the protein-coated oil droplets is the combination of electrostatic and steric repulsion. However, these emulsions are apt to flocculation when the magnitude of the repulsion forces between the droplets are reduced by high ionic strength in the suspension [8]. Similar results have been observed by Tan et al. [14], who reported due to the screening effect of salt, NaCl would diminish the electrostatic interactions between droplets.

### 2.4. Effect of Thermal Treatment on the Physical Properties of PSP-Stabilized Emulsions

**Figure 5:**
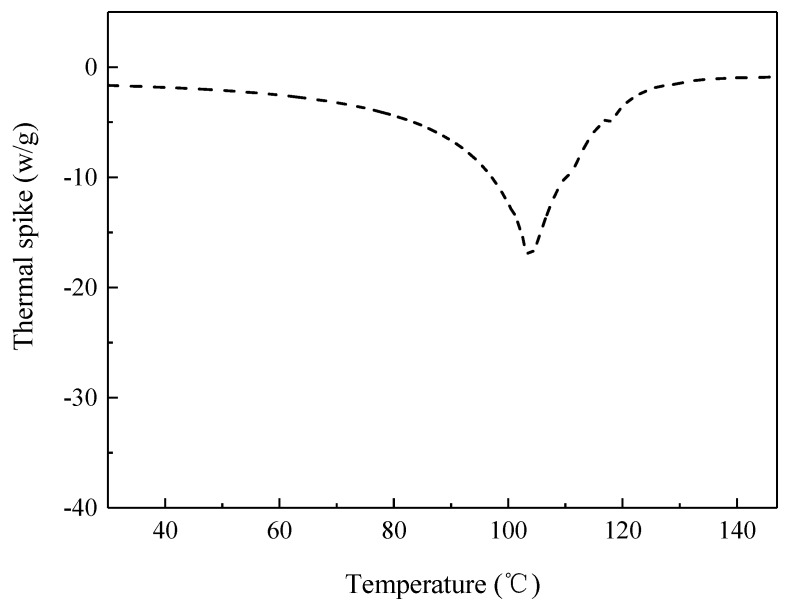
Differential scanning calorimetry (DSC) analysis of PSP.

Figure 5 is the differential scanning calorimetry (DSC) thermal curve of PSP in 1.0 wt % dispersion. As shown, the onset temperature (T_0_) and denaturation temperature (T_d_) of PSP were 83.12 °C and 103.52 °C, respectively. And the peak width at half height (ΔT_1/2_) of PSP was 20.40 °C.

**Figure 6:**
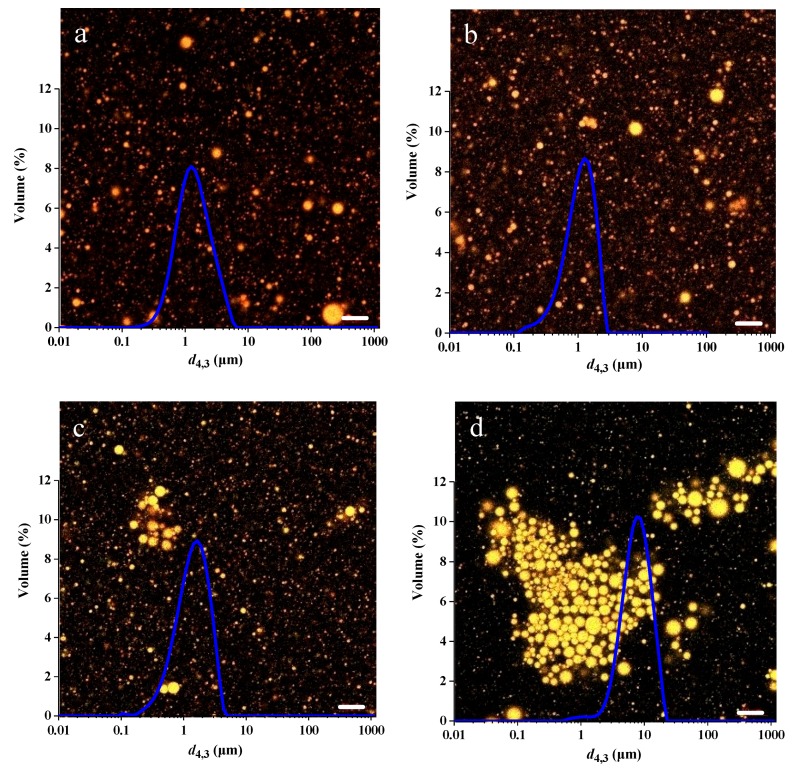
Influence of thermal treatment on the particle-size distribution and CLSM of PSP-stabilized emulsions. (**a**) the control (emulsion without thermal treatment), (**b**–**d**) emulsions with thermal treatment at 70 °C, 80 °C, 90 °C for 30 min, respectively. Scale bar in the micrographs corresponds to 10 μm. Values are means ± SDs (n = 3).

The effect of thermal treatment over a range of 70–90 °C on PSP-stabilized emulsions was investigated and the particle-size distribution and CLSM graphs are represented in Figure 6a–d. It can be observed that all the emulsions exhibited a conspicuous distribution peak but the extent and position of the conspicuous peak varied.

At 90 °C thermal treatment, the *d*_4,3_ of the emulsion was measured as 11.43 μm and extensive droplet aggregation can be observed in Figure 6d. A possible explanation for this phenomenon is that thermal denaturation of the adsorbed proteins [18]. Thermal denaturation and unfolding of adsorbed proteins on the droplet surfaces would result in the exposure of non-polar and sulfhydryl groups, thus increasing protein-protein interactions and causing droplet aggregation [10].

The *d*_4,3_ of the emulsion treated at 80 °C was determined as only 1.97 μm. It did not show an obvious droplet aggregation tendency as illustrated in Figure 6C. Moreover, when treated at 70 °C, the particle-size distribution was similar to the control sample (Figure 6a,b). Previous study has reported that a moderate thermal treatment and subsequent structural unfolding of proteins are benefit to their emulsifying properties [21]. Consequently, in the present study, the thermal treatment temperature of 70 °C should be favorable for the emulsifying property of PSP.

**Figure 7:**
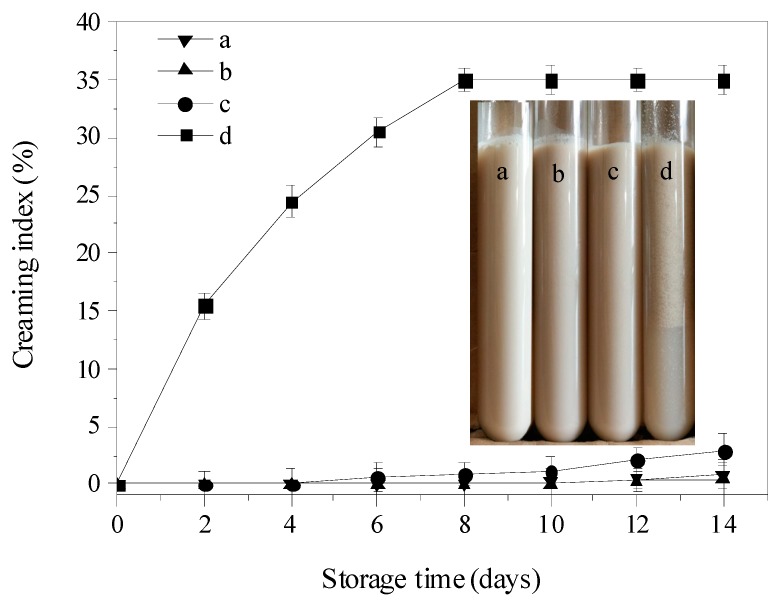
Influence of thermal treatment on the CI and creaming stability of PSP-stabilized emulsions. (**a**) the control (emulsion without thermal treatment), (**b**–**d**) emulsions with thermal treatment at 70 °C, 80 °C, 90 °C for 30 min, respectively. Values are means ± SDs (n = 3).

Figure 7 represents the influence of thermal treatment on the creaming index (CI) and creaming stability of different emulsions after 2 weeks storage. As time extended, emulsions a–c showed good storage stability and no clear phase separation was observed. However, for the emulsion treated at 90 °C (emulsion d), its creaming index gradually increased during storage and reached the maximum at the 8th day (35.12%). As illustrated, emulsion d exhibited a clear layer separation with an obvious boundary between the cream and serum layers. Figure 5 indicates the onset denaturation temperature of PSP was at 83.12 °C. Therefore, a poor stability was observed in emulsion d because the PSP had denatured at 90 °C thermal treatment.

## 3. Materials and Methods

### 3.1. Materials

Defatted perilla seed meal and perilla seed oil were purchased from Changbai-Workshop Co. Ltd. (Jilin, China). Nile blue and Nile red of technical grade were acquired from Sigma-Aldrich Chemical Co. Ltd. (St. Louis, MO, USA). Deionized water was used and all other reagents were of analytical grade.

### 3.2. Perilla Seed Protein (PSP) Preparation

PSP was extracted from defatted perilla seed meal according to Sarkar and Kaul’s method [22] with slight modifications. The perilla seed meal was ground using a pulverizer with screen size of 0.9 mm to obtain a fine powder. Perilla seed powder (80 g) was dispersed in 800 mL of 0.5 mol/L NaCl solution (adjusting pH to 8.0) and stirred at 50 °C for 1 h. The resultant suspension was centrifuged at 12,000× *g* for 30 min using Avanti J-26s centrifuge (Beckman Coulter, Brea, CA, USA) and the supernatant was collected. Then 0.1 mol/L HCl was used to adjust the pH of the supernatant to the isoelectric point (pH 4.5) and the supernatant was centrifuged at 12,000× *g* for 30 min. The precipitate obtained was washed with distilled water and redissolved with pH adjusted to 7.0. Afterwards, it was dialyzed against distilled water at 4 °C for 48 h. An Alpha 1-4 LD plus freeze-dryer (Marin Christ, Osterode, Germany) was used to lyophilize the solution. The obtained PSP had a protein content of 85.35% as determined by Kjeldahl nitrogen determination method.

### 3.3. Protein Solubility at Different pH Levels

Protein solubility of PSP was measured by dispersing the PSP samples in deionized water to acquire a final solution of 0.2 wt % in protein. The pH of the PSP dispersion was adjusted from 10.0 to 1.0 and then it was centrifuged at 12,000× *g* for 30 min (25 °C). The content of protein for the resultant dispersion was determined by the Lowry method [23].

### 3.4. Phase Diagram

Protein dispersions containing 1.0 wt % PSP at various NaCl concentration (0, 50, 100, 150, 200, 250, 300, 350, 500 mM) were prepared. The pH of the dispersions was adjusted to values at the range of 11.0–2.0. Then the dispersions were stored at room temperature overnight. Through visual observation, the solubility of the systems was evaluated as clear, cloudy, little precipitate, precipitate, respectively. Therefore, a phase diagram of pH against NaCl concentration can be constructed.

### 3.5. DSC Analysis

DSC analysis of PSP was performed using a TA Q200-DSC thermal analyzer (TA Instruments, New Castle, DE, USA). PSP dispersion (1.0 wt %) were placed into an aluminum pan and then sealed the aluminum pan. A sealed empty pan was used as contrast. The parameters were set as follows, temperature scan range: 0–150 °C; temperature rate: 5 °C/min; flow rate of nitrogen: 50 mL/min.

### 3.6. Emulsion Preparation

The PSP dispersion (0.25–1.5 wt %) was prepared in 10 mmol/L phosphate buffer at pH 7.0 by stirring for 2 h at 25 °C. The dispersion was refrigerated at 4 °C for 24 h to ensure complete hydration of the protein. A coarse emulsion was prepared by mixing PSP dispersion (90.0 wt %) and perilla seed oil (10.0 wt %). The mixture of perilla seed oil and protein dispersion was pre-emulsified using an Ultra-Turrax T10 basic disperser (Ika-Werke, Staufen, Germany) at 15,000 rpm for 2 min. The mixture was then homogenized for two passes at 60 MPa and 15 MPa, respectively using a ATS-Basic I high pressure homogenizer (ATS Engineering Ltd., Toronto, ON, Canada). Sodium azide (0.02 wt %) was used to prevent microbial growth during storage of the emulsions.

### 3.7. Particle-Size Distribution and Volume-Average Diameter

The particle-size distribution and volume-average diameter (*d*_4,3_, μm) of emulsions were measured by using a Malvern MasterSizer 2000 (Malvern Instruments Co. Ltd., Worcestershire, UK). The refractive index, the adsorption of dispersed phase and the refractive index of continuous phase were set at 1.414, 0.001 and 1.330, respectively [24].

### 3.8. ζ-Potential Measurement

The emulsion sample was diluted with 5 mM phosphate buffer (pH 7.0, sample dilution 1:1000) and then transferred into a Model DTS 1060C zeta cell (Model DTS 1060C, Malvern Instruments Co. Ltd., Worcestershire, UK). A Malvern Zetasizer Nano ZS instrument (Malvern Instruments Co. Ltd., Worcestershire, UK) was used to measure the ζ-potential of emulsions at 25 °C. The equilibrium time was 30 s.

### 3.9. Interfacial Protein Concentration

Interfacial protein concentration of emulsions was determined according to Long et al.’s method [25] with some modifications. 1 mL of each emulsion sample was placed into a centrifuge tube and centrifuged at 10,000× *g* for 30 min using Avanti J-26s centrifuge. After centrifuging, the interfacial protein content of cream layers was determined by Kjeldahl nitrogen determination method Interfacial protein concentration (*Γ*, mg/m^2^) was calculated by Equation (1).

Interfacial protein concentration (*Γ*) = (interfacial protein content/interfacial area) × 1000 (1)

where the interfacial area was measured using the Mastersizer 2000.

### 3.10. Confocal Laser Scanning Microscopy (CLSM)

A Leica TCS SP2 confocal laser scanning microscope (Leica, Heidelberg, Germany) was used to obtain the CLSM graphs of emulsion samples in fluorescence mode. The mixture of 0.02% (*w*/*v*) Nile red (labeling lipid) and 0.1% (*w*/*v*) Nile blue (labeling protein) were used as staining solution. Aliquots of 40 μL staining solution was added to 1 mL of emulsion with slight stirring. The stained emulsion was placed onto a glass slide and then covered with a cover glass slowly. Silicone oil can be smeared around the cover glass to avoid the evaporation of moisture. A 100× oil-immersion lens was used. The fluorescence in the samples was excited with the Argon Krypton laser (488 nm) and the Helium Neon laser (633 nm).

### 3.11. Visual Observation of Creaming and Determination of Creaming Index

Each sample was transferred into a glass test tube (1.5 cm internal diameter × 15 cm height) of ten milliliters (10 mL). During two weeks of storage, the extent of creaming was evaluated using creaming index [26], which was defined as CI = 100% × (H_S_/H_E_) for the serum layer, where H_E_ is the total height of the emulsion and H_S_ is the height of the subnatant (turbid) serum layer.

### 3.12. Stability of Emulsions

The pH, salt and thermal stabilities of emulsions were examined by altering their environments. And several stability indexes mentioned above were determined.

#### 3.12.1. pH Stability

The pH of emulsions was adjusted to values ranging from 9.0–3.0 by adding either HCl or NaOH to create a series of samples. Then these emulsions were stirred using a magnetic stirrer at 600 rpm for 30 min.

#### 3.12.2. Salt Stability

Different amounts of NaCl were added to the fresh emulsions to get the desired NaCl concentrations (50–350 mmol/L). Then these emulsions were stirred using a magnetic stirrer at 600 rpm for 30 min to ensure they were homogeneous.

#### 3.12.3. Thermal stability

The fresh emulsions were incubated in water bath at 70 °C, 80 °C and 90 °C respectively, for 30 min and then cooling them to room temperature by putting them in an ice water bath. Emulsion without any thermal treatment was set as the control.

### 3.13. Statistical Analysis

All the data in the present study were obtained from the mean values of three measurements. A one-way ANOVA was performed to analyze the significant difference between samples (*p* < 0.05).

## 4. Conclusions

This study examined the influences of protein concentration and environmental stresses on the physical properties of PSP-stabilized O/W emulsions. Results showed that increasing PSP concentration (up to 1.0 wt %) was favorable for the emulsions against aggregation/flocculation. PSP-stabilized emulsions were stable except at pH values close to their isoelectric point (pH 4.5). Emulsions exhibited good stability at NaCl concentration less than 150 mmol/L. Excellent thermal stability was observed in emulsion with a thermal treatment of 70 °C. These results suggest that PSP is potential to be applied in emulsion-based food systems as an emulsifier.

## Figures and Tables

**Table 1:** Emulsion characteristics, including *d*_4,3_, ζ-potential and interfacial protein concentration (*Γ*) of emulsions with different protein concentration.

PSP Concentration	*d*_4,3_ (μm)	ζ-Potential (mV)	*Γ* (mg/m^2^)
0.25 wt %	5.54 ± 0.07 ^a^	−18.62 ± 0.08 ^a^	0.65 ± 0.04 ^a^
0.5 wt %	4.87 ± 0.08 ^b^	−26.59 ± 0.07 ^b^	0.86 ± 0.05 ^b^
1.0 wt %	1.27 ± 0.03 ^c^	−48.97 ± 0.10 ^c^	1.55 ± 0.05 ^c^
1.5 wt %	1.18 ± 0.02 ^c^	−49.34 ± 0.08 ^d^	1.63 ± 0.03 ^c^

Values are means ± SDs (n = 3); Values of the same series, with the different letters (a, b, c, d) are significantly (*p* < 0.05) different.
